# Artemether-lumefantrine and liver enzyme abnormalities in non-severe *Plasmodium falciparum* malaria in returned travellers: a retrospective comparative study with quinine-doxycycline in a Portuguese centre

**DOI:** 10.1186/s12936-017-1698-y

**Published:** 2017-01-25

**Authors:** André Silva-Pinto, Rogério Ruas, Francisco Almeida, Raquel Duro, André Silva, Cândida Abreu, António Sarmento

**Affiliations:** Infectious Diseases Department, Centro Hospitalar São João, Alameda Professor Hernani Monteiro, 4200 Porto, Portugal

**Keywords:** Malaria, Artemether–lumefantrine, Liver dysfunction, *Plasmodium falciparum*

## Abstract

**Background:**

Artemisinin-based therapy is the current standard treatment for non-severe malaria due to *Plasmodium falciparum.* The potential for asymptomatic liver toxicity of this therapy and its implication in clinical practice is currently unknown. The aim of this study is to assess the hepatic function in patients treated with a standard three-day artemisinin-based regimen and to compare it with the quinine-doxycycline regimen.

**Methods:**

Retrospective and comparative study of returned adult travellers admitted with non-severe *P. falciparum* malaria. Fifty-seven patients were included: 19 treated with artemisinin-based therapy and 38 with quinine-doxycycline therapy.

**Results:**

During treatment, when compared with quinine-doxycycline group, the artemisinin-lumefantrine group presented a higher proportion of significant liver enzyme abnormalities (42 vs. 5%, p < 0.01) and a higher peak value of aspartate aminotransferase (131 vs. 64 U/L, p < 0.01) and alanine aminotransferase (99 vs. 75 U/L, p = 0.05). None of the patients was symptomatic, there were no treatment interruptions and all patients achieved clinical cure.

**Conclusions:**

Treatment of uncomplicated falciparum malaria with artemisinin-based therapy might cause asymptomatic liver enzyme abnormalities in the first days of treatment. Nevertheless, these liver enzyme abnormalities seem to be harmless, asymptomatic and self-limited.

## Background

Artemisinin based combination therapy (ACT) is the current standard treatment for uncomplicated malaria caused by *Plasmodium falciparum* [1]. Owing to their rapid onset of action and quick elimination, artemisinin derivatives are very effective in reducing parasite load and have favourable safety and tolerability profiles [2, 3]. In Portugal, artemether/lumefantrine fixed dose combination (AL), currently the only available ACT, came into medical use in 2013 and is an alternative to quinine plus doxycycline (QD), the previous first-line regimen [1]. The main reasons for this paradigm change are the safety and tolerability profiles of AL and the remaining ACT regimens compared to QD [3, 4].

Hepatotoxicity associated with ACT has been described in animal models [5–7] and several series in humans have shown elevation of liver enzymes of undetermined clinical significance [8]. On the other hand, it has rarely been described for QD [9–11] and AL regimens [12]. Even pooled analysis of randomized controlled trials with AL did not find significant hepatic toxicity. However, the laboratory analysis was performed only at baseline and on day 28 the AL effect in the acute phase was not tested [13].

The potential for asymptomatic liver toxicity and its implication in clinical practice need clarification in order to understand the possible impact of ACT on a wider scale. It is also important to assess whether transaminase elevation seen during ACT can be explained solely by malaria-associated liver damage or if it is more likely associated with drug toxicity. It is important to take into account that most ACT for malaria treatment takes place in low-resource settings, where routine toxicity monitoring might not be feasible.

The aim of this study is to assess the hepatic function in patients hospitalized with non-severe falciparum malaria treated with a standard three-day regimen of AL, and to compare them with a historical cohort of patients admitted for the same reason and treated with a QD regimen.

## Methods

A retrospective, comparative study was performed, including adult patients (over 18 years old) admitted to the Infectious Diseases Ward of Centro Hospitalar de São João, Porto, Portugal with the diagnosis of non-severe falciparum malaria (as defined by the World Health Organization [1]). The diagnosis of malaria was based on rapid diagnostic test (BINAX Malaria Now^®^) and microscopy (thin blood film; parasitaemia is expressed as % of parasitized red blood cells). The liver function tests were performed with COBAS^®^ and the same technique was used in both groups [kinetic enzyme essay for transaminases and colorimetric enzyme essay for bilirubin, gamma-glutamyl transferase (GGT) and alkaline phosphatase (AP)]. Patients were identified patients using the hospital database (IEG-HSJ^®^) and subsequently clinical records were consulted. Data concerning demography (gender, age, nationality), past medical history, country of malaria acquisition, duration of symptoms, date of travel to Portugal, duration of hospitalization, duration of symptoms after beginning of treatment, co-infections, level of parasitaemia and its trend, level of liver enzymes before treatment and its highest value, coagulation times, symptoms of liver dysfunction (asthaenia, nausea, vomiting, icterus) and death was collected. The aim was to compare the liver dysfunction in patients hospitalized with non-severe falciparum malaria treated either with AL or QD. Two groups of patients were created according to the treatment received: those treated with AL (all patients admitted during 2014) and those treated with QD *per os* (all patients admitted between 2010 and 2013), with an AL: QD ratio of 1: 2. The primary outcome was significant liver enzyme abnormalities (defined as an elevation of trasaminases more than five times the normal upper limit in asymptomatic patients or three times in symptomatic patients; elevation of total bilirubin or AP more than two times the normal upper limit). The secondary outcomes were malaria cure, treatment interruption due to side effects and normalization of liver enzymes on follow-up. The most appropriate measure of central tendency and distribution for variables description (mean and standard deviation if normal distribution or median and interquartile range if non-normal distribution) was used. The two groups were compared using the most suitable statistical test; statistical analysis was performed with SPSS 22.0^®^ and a two-sided *p* value <0.05 was considered statistically significant.

The protocol was approved by the Centro Hospitalar São João Ethics Committee.

## Results

 A total of 57 patients was included: 19 treated with AL and 38 with QD. The main epidemiological, clinical and analytical variables considered are shown in Table 1, including the comparison of the two groups. There were no statistically significant differences between the two groups regarding the proportion of males or the mean age (p = 0.65 and p = 0.48, respectively). The majority of patients were Portuguese and acquired malaria in Angola; 49.3% had a previous episode of malaria. None of the patients reported previous hepatic disease or anti-malarial prophylaxis use.

**Table 1:** Main epidemiological, clinical and analytical variables comparing the two treatment groups: artemether–lumefantrine (AL) and quinine-doxycycline (QD)

Characteristic	Total (n = 57)	AL group (n = 19)	QD group (n = 38)	*p* value
Demographics				
Male gender (n, %)	51 (89%)	18 (95%)	33 (87%)	0.65^a^
Age (mean in years, SD)	41.2 (9.7%)	40 (10)	42 (10)	0.63^b^
Portuguese nationality (n, %)	49 (86%)	18 (95%)	31 (81%)	0.25^c^
Country of malaria acquisition				0.48^c^
Angola (n, %)	37 (65%)	11 (58%)	26 (68%)	
Mozambique (n, %)	7 (12.3%)	2 (11%)	5 (13%)	
Other (n, %)	13 (22.8%)	6 (31.6)	7 (18.4%)	
Past history of malaria (n, %)	23 (49.3%)	5 (26%)	18 (47%)	0.16^c^
Parasitaemia				
Initial parasitaemia (n, %) (median, IQR)	1.0 (1.5)	0.5 (0.9)	1.0 (1.5)	**0.03** ^d^
Days until symptoms resolution (median, IQR)	2 (2)	2 (1)	2 (2)	0.89^d^
Days until no parasitaemia (median, IQR)	2 (2)	2 (2)	3 (3)	0.09^d^
Liver panel^e^				
At diagnosis				
Initial AST (median, IQR)	44 (29.5)	46 (41)	42 (26)	0.99^d^
Initial ALT (median,IQR)	41 (52)	32 (52)	44 (53)	0.50^d^
Initial GGT (median, IQR)	51 (91)	48 (100)	52 (87)	0.84^d^
Initial AP (median, IQR)	67 (32.5)	77 (32)	60 (30)	0.13^d^
Initial total bilirubin (median, IQR)	1.2 (0.7)	1.2 (0.7)	1.2 (0.7)	0.53^d^
Initial aPTT (median, IQR)	34.8 (5.9)	33 (5)	34 (7)	0.45^d^
During treatment				
Highest AST (median, IQR)	64 (83)	131 (278)	58 (50)	**<0.01** ^d^
Number of days until highest value (median, IQR)	2 (4)	3 (3)	1 (5)	0.43^d^
Highest ALT (median, IQR)	83 (88.5)	99 (297)	75 (72)	**0.05** ^d^
Number of days until highest value (median, IQR)	3 (4)	3 (2)	2.5 (4)	0.77^d^
Highest GGT (median, IQR)	72.5 (108)	93 (160)	66 (99)	0.16^d^
Highest AP (median, IQR)	71 (30)	84 (63)	70 (31)	0.07^d^
Highest total bilirubin (median, IQR)	1.6 (1.3)	1.6 (1.4)	1.5 (1.3)	0.85^d^
Highest aPTT (median, IQR)	34.8 (6.2)	38 (6)	34 (5)	0.06^d^
Outcomes				
Asymptomatic liver dysfunction (n, %)	10 (17.5%)	8 (42%)	2 (5%)	**<0.01** ^**c**^
Symptomatic liver dysfunction (n,%)	0	0	0	–
Death (n, %)	0	0	0	–

Bold indicates significant values at *p* ≤ 0.05

^a^Pearson Chi square

^b^Independent *t* test

^c^Fisher’s exact test

^d^Mann–Whitney *U* test

^e^Reference laboratory laboratories: AST 10–37 U/L; ALT 10–37 U/L; GGT 10–49 U/L; AP 30–120 U/L; total bilirubin <1.2 mg/dL; aPTT 24.2–36.4 s

The initial parasitaemia was significantly higher in the QD group (median 1.0 vs. 0.5; p = 0.03, Mann–Whitney *U* test). There was no difference in the number of days until symptom resolution, although the number of days until parasitaemia clearance was higher in the QD group (but not statistically significant, p = 0.09).

Other infectious causes of liver dysfunction were excluded.

At diagnosis, there were no differences between the groups considering the levels of aspartate aminotransferase (AST), alanine aminotransferase (ALT), GGT, AP, total bilirubin or activated partial thromboplastin time (aPTT). However, during treatment, the AL treatment group presented a higher proportion of patients with significant liver enzyme abnormalities [8/19 (42%) vs. 2/38 (5%); p < 0.01] and a higher peak value of AST and ALT (p < 0.01 and p = 0.05, respectively). The remaining liver panel was not significantly different between the groups. None of the patients was symptomatic and there were no treatment interruptions. In the AL-treated group, the duration of hospitalization was not prolonged in those with hepatic dysfunction (median: 5.5 vs. 4 days; p = 0.06, Mann–Whitney *U* test). All patients were discharged with clinical and microbiological evidence of cure; the mean duration of hospitalization was 5.6 days. AST and ALT returned to normal during outpatient follow-up in all cases.

## Discussion

In this study, it is reported for the first time the association between AL treatment in non-severe falciparum malaria and liver enzyme abnormalities occurring on the first day of treatment (which was not observed in the QD group). Treatment of uncomplicated malaria caused by *Plasmodium falciparum* with ACT has high success rates and low described toxicities [13]. However, as for most diseases of the underdeveloped world, post-marketing surveillance is scarce, especially for non-fatal complications.

When AL treatment became the standard treatment in Centro Hospitalar São João, a new trend towards an elevation of hepatic transaminases was noted that could raise questions about AL treatment safety. Nevertheless, these results do not support this view, as significantly higher ALT and AST observed in patients treated with AL were not accompanied by symptoms or complications related to hepatic dysfunction and did not delay hospital discharge.

Hepatic (elevation of AST, ALT and GGT; liver cells degeneration on histology), cardiac, renal, and neurotoxicity, as well as embryonic malformations, associated with ACT regimens have been described in animal models [5–7, 14]. However, these have not been shown to be relevant in widespread clinical use in humans. This discrepancy in toxicity between human and animal studies is thought to be explained by pharmacokinetic properties of artemisinins. Toxicity seems to be associated mostly with drug exposure (i.e., the area under the drug concentration–time curve). Intramuscular administration of artemisinins, mostly used in animal experiments, seems to be associated with delayed drug release and is thus more prone to toxicity, as opposed to oral administration used to treat human disease, which seems to be generally safe [15, 16].

In the setting of no previous hepatic disease (as in this study), hepatic dysfunction in the context of non-severe malaria can either be due to the disease or to drug toxicity. The only statistically significant difference between the groups (AL vs. QD) regarding general characteristics and disease presentation was the level of parasitaemia, which was significantly higher in the QD group. As such, in this study, hepatic dysfunction caused by malaria does not seem a plausible explanation. On the other hand, there is no data assessing hepatic toxicity during the first days of treatment [13]. Liver damage associated with ACT seems to arise from the release of reactive oxygen species and oxidative stress resultant from their strong parasitical activity, with this abrupt and marked change in oxidative status having the potential of damaging liver cells by decreasing the hepatic glutathione S-transferase levels and the superoxide dismutase and catalase activities [17, 18]. However, for the vast majority of patients in all reviews and citations, as well as for all patients included in this comparative study, these alterations are quite benign and always transient, most likely translating a physiological, short-term, hepatic adaptation to artemisinin derivatives rather than true toxicity [13, 19]. On the other hand, the onset of action between QD and AL is known to be different [1, 20]: the latter combination has a quicker onset of action, with a higher per cent of parasite killing (and hence a quicker drop in the level of parasitaemia). The authors question if this faster parasite killing, with consequent higher load for clearance by the reticuloendothelial system, would explain self-limited liver enzyme abnormalities.

The main limitation of this study, regarding its purpose, is that it is not a randomized controlled trial. Because this were carried out prior to the introduction of ACT in clinical practice in Portugal, the switch from QD to AL was automatic when the regimen was available. The differences between QD and AL were suspected with the continued use of the latter. However, previous studies were not designed to find a difference, as described in this study.

This study is a small, retrospective, observational study and therefore selection bias might exist: the QD-treated patients were selected in 2010–2013 and the AL-treated patients in 2014. However, the migration pattern to Portugal did not change during the study period (2010–2014) and the baseline demographic and analytic characteristics did not differ between the groups.

## Conclusions

Treatment of uncomplicated falciparum malaria with AL might cause asymptomatic liver enzyme abnormalities in the first days of treatment. However, the liver enzyme abnormalities seem to be harmless, asymptomatic and self-limited, not affecting clinical outcome. As such, this should not discourage the use of this treatment, but clinicians should be aware of this potential effect in order not to overestimate or undervalue it.
